# Bone quality, mineral density, and fractures in heart failure

**DOI:** 10.1371/journal.pone.0293903

**Published:** 2023-11-03

**Authors:** Andre Luiz Canteri, Luana Bassan Gusmon, Cesar Luiz Boguszewski, Victoria Zeghbi Cochenski Borba

**Affiliations:** 1 Division of Cardiology, Department of Internal Medicine, Hospital de Clínicas, Federal University of Paraná, Curitiba, Paraná State, Brazil; 2 Health Sciences and Internal Medicine Postgraduation Department, Federal University of Paraná, Curitiba, Paraná State, Brazil; 3 Endocrine Division (SEMPR), Department of Internal Medicine, Federal University of Paraná, Curitiba, Paraná State, Brazil; Federal University of Minas Gerais, BRAZIL

## Abstract

**Background:**

The trabecular bone score (TBS) indirectly estimates bone quality and predicts low-impact fractures independently of bone mineral density (BMD). However, there is still a paucity of data linking bone and heart diseases, mainly with gaps in the TBS analysis.

**Methods:**

In this cross-sectional study, we evaluated TBS, BMD, and fractures in patients with heart failure with reduced ejection fraction (HFrEF) and in sex-, BMI- and age-matched controls, and we assessed the fracture probability using the FRAX tool, considering active search for fractures by vertebral fracture assessment (VFA) and the adjustment for the TBS.

**Results:**

TBS values were 1.296 ± 0.14 in 85 patients (43.5% women; age 65 ± 13 years) and 1.320 ± 0.11 in 142 controls (*P* = 0.07), being reduced (< 1.31) in 51.8% and 46.1% of them, respectively (*P* = 0.12). TBS was lower in patients than in the controls when BMD was normal (*P* = 0.04) and when the BMI was 15–37 kg/m^2^ (*P* = 0.03). Age (odds ratio [OR] 1.05; *P* = 0.026), albumin (OR 0.12; *P* = 0.046), statin use (OR 0.27; *P* = 0.03), and energy intake (OR 1.03; *P* = 0.014) were associated with reduced TBS. Fractures on VFA occurred in 42.4% of the patients, and VFA and TBS adjustment increased the fracture risk by 16%-23%.

**Conclusion:**

Patients with HFrEF had poor bone quality, with a better discriminating impact of the TBS assessment when BMD was normal, and BMI was suitable for densitometric analysis. Variables related to the prognosis, severity, and treatment of HFrEF were associated with reduced TBS. VFA and TBS adjustment increased fracture risk.

## Introduction

Heart failure (HF) and osteoporosis (OP) are prevalent diseases, whose incidence increases with age. They significantly affect the health care system, and predispose sufferers to disability, loss of independence, hospitalizations, and impaired quality of life. They share other common risk factors such as smoking, alcohol use, and low level of physical activity, and comorbidities such as renal insufficiency, diabetes, vitamin D deficiency, hyperparathyroidism, and obesity [1]. Moreover, cardiovascular diseases are highly prevalent at the time of OP diagnosis, and HF is an independent risk factor for osteoporotic fractures [2, 3]. Also, the hyperactivation of the renin–angiotensin–aldosterone system, the production of inflammatory mediators, the osteoprotegerin system and the use of drugs for HF could affect the skeleton [4]. On the other hand, OP and previous fragility fractures are risk factors for HF [5].

OP is characterized by a reduction in bone mineral density (BMD), microarchitectural deterioration, reduced bone strength, and predisposition to fractures [6]. The low degree of chronic inflammation that occurs in systemic diseases can cause bone loss through neuroendocrine stimuli [1, 7]. The high levels of aldosterone, in addition to the hyponatremia that is sometimes present in HF, could exacerbate bone damage [8, 9].

There is little information linking HF and OP in the literature. Between 25% and 60% of patients with severe heart disease evaluated before heart transplantation might present low BMD [10, 11], with approximately 1% bone loss per year [2, 12]. Patients with HF have lower BMD than non-HF patients, and it has been positively correlated with worse functional capacity, left ventricular ejection fraction, and vitamin D levels. Conversely, it has been inversely correlated with parathyroid hormone levels and frailty status [12–14]. Furthermore, some medications used in the treatment of cardiac diseases, such as anticoagulants and loop diuretics, may worsen bone loss [15, 16]. In addition, the presence of atrial fibrillation, common in HF, has been associated with fragility fractures [17].

Trabecular bone score (TBS), assessed by lumbar spine densitometry, is a simple and inexpensive way to estimate the quality of bone indirectly. It is a textural index that correlates with the microarchitectural damage of the bone and predicts the occurrence of low-impact fractures independently of BMD [18]. In HF with reduced ejection fraction (HFrEF), 55% of heart transplant patients had impaired or partially impaired TBS, with reduction over time after transplantation. Low TBS was also associated with age, higher body mass index (BMI), and low BMD, and although most fractures in these patients occurred with osteopenia, no association between degraded TBS and the presence of vertebral fractures (VFs) has been described [19].

As far as we know, researchers have not evaluated TBS’s role exclusively in untransplanted patients with HFrEF under optimal treatment. Therefore, the aims of this study were to evaluate TBS, BMD, and fractures, as well as the likelihood of future fractures considering the analysis of TBS and the active search for fractures in a selected sample of patients with HFrEF who did not have an indication of heart transplant and who were stable under optimal HF evidence-based guideline treatment compared with controls.

## Material and methods

### Data sources, study design and population

This is a cross-sectional, controlled study approved by the Ethics Committee on Human Research of our institution in accordance with the ethical standards (approval number: 1.314.289; CAAE: 48506015.2.0000.0096).

Men and women over 18 years of age with HFrEF attending the cardiology outpatient HF clinic were selected from the echocardiography database of our institution and constituted the HFrEF group (HFrEFG). All participants underwent clinical, physical, and laboratory evaluations in accordance with the research protocol between November 2015 and December 2017 and signed the informed consent form after adequate verbal explanation and after reading the informed consent form. All participants received a signed copy of this document. Patient data were collected individually under direct consultation, and to avoid biases related to the identification of participants in the data analysis, the names and any data that could identify the research participants were coded and excluded from the analysis worksheet.

Excluded from this study were patients who had clinical intercurrences or hospitalization 90 days prior to the protocol; those who had at least moderate cardiac stenotic valvar disease, chronic obstructive pulmonary disease (COPD) with FEV1 < 50%, interstitial lung diseases, pregnancy, current daily alcohol intake > 25 g, neuromuscular or orthopedic limitations, and chronic kidney disease with clearance ≤ 30 ml/min. 1.73 m^2^; patients with type 1 diabetes; patients with type 2 diabetes (DM2) with glycated hemoglobin (HbA1C) ≥ 8.5%, with severe microvascular chronic complications, or using 2 or more hypoglycemic drugs or insulin therapy; and any patients with other known systemic diseases and/or taking medications that could directly affect bone health and body composition.

On the day of the research protocol evaluation, a fasting blood sample was collected from all participants, medical records were revised, and questionnaires to provide clinical and demographic data, cardiac functional capacity, and levels of physical activity, ground-level falls, and clinical low-impact fractures were distributed. Densitometry and nutritional assessment were also conducted on the same day. The whole research protocol was fully described elsewhere [20, 21], and the main evaluations are briefly described below.

#### Anthropometric evaluation and physical exam

Body weight was measured with barefoot patients wearing underwear, and height was measured on a vertical scale. Edema was graded in 5 progressive categories (0 to 4+) after inspection and palpation of the lower limbs. Patients with 2+ edema were excluded.

#### Physical activity questionnaire

Physical activity was evaluated using the International Physical Activity Questionnaire-short version (short-IPAQ) validated for Portuguese and applied as a structured interview. We categorized patients as sedentary (no physical activity for at least 10 continuous minutes/week), insufficiently active (physical activity for at least 10 continuous minutes/week), or active (vigorous physical activity for at least 20 minutes/session for at least 3 days/week, moderate activity or walking at least 5 days/week for at least 30 minutes/session, or any combination activity with a total duration at least 150 minutes/week, 5 or more days/week).

#### Comorbidity evaluation

The Charlson Comorbidity Index (CCI) was used, and all patients were considered as having HF (minimal score = 1) [22].

#### Strength evaluation

Handgrip strength (HGS) was measured bilaterally with a Charder® MG 4800 Medical Handgrip dynamometer, 3 times on each side with a 1-minute interval between each evaluation, alternating sides. Weakness was defined as HGS < 26 kg or HGS adjusted for BMI < 1 in men, and < 16 kg or < 0.56 in women. The highest admeasurement was used.

#### Physical performance—Gait speed

A gait speed test was conducted in a central flat corridor 4 m in length with 1-m additional acceleration and deceleration zones at each end. The time required to complete the 4-m path was the arithmetic mean of three concordant evaluations. A gait speed of ≤ 0.8 m/s was considered low gait velocity.

#### Laboratory exams

The ARCHITECT C 8000 analyzer, Abbott®, was used for almost all plasma biochemical analyses, which were conducted on the day of the protocol evaluation. Serum levels of 25-hydroxyvitamin D (25OHD) were determined by immunochemiluminescence (LIAISON®). HbA1C (%), total cholesterol (mg/dL), HDL (mg/dL), triglycerides (TG-mg/dL), INR (international normalized ratio), and blood count (XN-3000, Sysmex®) were analyzed retrospectively.

#### Densitometry

All participants were submitted to a total body densitometry by dual X-ray absorptiometry (DXA) using Lunar Prodigy equipment (GE Medical Systems, Madison, WI, USA) and the Encore program. Total mass, total lean mass (TLM), total fat mass, trunk fat mass, and the appendicular lean mass (ALM), which is the sum of lean mass of the arms and legs, were evaluated. Low ALM was defined as < 19.75 kg in men and < 15.02 kg in women.

BMD scans were evaluated in the spine (L1-L4), femoral neck, and total hip and their results expressed in g/cm^2^. The results were analyzed according to the World Health Organization (WHO) and the International Society for Clinical Densitometry (ISCD). BMD was classified as normal, osteopenia (T score < −1.0 and > −2.5 DP), OP (T score ≤ 2.5 DP), or low bone mass for patient age (Z score < −2.0 DP) when the patients were younger than 50 or premenopausal. Also, BMD results were stratified in normal (T score ≥ 1 or Z score ≥2) or low (presence of osteopenia, OP, or BMD below that expected for the age range).

TBS was obtained from DXA lumbar scans using TBS iNsight version 3.0.2.0 (MediMaps, Geneva, Switzerland). The microarchitecture was considered degraded when values were ≤ 1.230, partially degraded when values were > 1.23 and < 1.31, and normal when they were ≥ 1.31 [18]. In this article, reduced TBS refers to degraded or partially degraded TBS. Considering that diabetes and obesity may influence TBS [18, 23], a separate analysis was conducted of TBS excluding all participants with diabetes and those with BMI < 15 or ≥ 37 kg/m^2^.

#### Vertebral fracture assessment (VFA)

A search for fractures of the spine was performed by VFA in the patients with HFrEF (HFrEFG). A blinded skilled radiologist analyzed fractures using Genant’s semiquantitative assessment [24]. T4 through L4 vertebrae images were analyzed, and nonvisible vertebrae were excluded.

#### Low-impact fractures

Low-impact fractures were defined as fractures resulting from a fall from standing height or less, at axial (ribs, thoracic, or lumbar vertebrae) or appendicular (forearm, humerus, and femur) skeletal sites caused by a trauma not sufficient to fracture a normal bone and were evaluated through direct inquiry and review of the patient’s medical records.

#### Fracture risk probability–Fracture Risk Assessment Tool (FRAX)

The 10-year probability of major fractures and hip fractures was estimated using the FRAX, which was adapted and developed for the Brazilian population [25]. FRAX fracture probability was adjusted for TBS [26], and the previous fracture was included in the tool when revealed in clinical history or by their presence on VFA.

#### Nutritional evaluation

Nutritional assessment was conducted through a 24-hour recall questionnaire. Analysis of the composition of the nourishment and meals was conducted by crossing the data obtained in the Brazil Nutri software with the table of nutritional composition of foods consumed in Brazil. Adequate intake was defined as ingested values between 90% and 110% of the established nutritional goal. The goals for protein intake were 2.0 g/kg/day for BMI > 30 kg/m2, 1.2 g/kg/day for > 65 years old, and 15% of the daily energy requirement for adults nonobese and ≤ 65 years old. For calcium, a minimum intake of 1000 mg/day was considered adequate, and for vitamin D, the minimum was 15 μg/day (< 70 years old) or 20 μg/day (> 70 years old).

### Control group (CG)

The CG comprised non-athlete individuals who were selected from a database of the Endocrine Division (SEMPR) of our institution, were clinically stable, and had no uncontrolled chronic diseases. They were matched according to age, sex, and BMI, and excluded were individuals with chronic severe or untreated systemic diseases, as well as those with uncontrolled diabetes, presenting with macro- or microvascular lesions or using insulin. The CG was submitted to the same evaluations under the same methodology as the HFrEFG, except for VFA, nutritional, and gait speed assessments.

### Statistical analysis

We used Stata/SE v14.1. (StataCorpLP, USA) for the analysis. Shapiro Wilk and/or Komorogov-Smirnov tests were conducted to assess the sample´s normality. Data are presented in absolute and relative frequencies for qualitative variables and mean ± SD or median (minimum and maximum) for quantitative variables. To compare the quantitative variables, Student’s t test or Mann–Whitney tests were conducted. The Kruskal–Wallis and post hoc tests were conducted for comparison of 3 or more groups, and the Fischer exact test or χ2 test was used for qualitative analysis. A multivariate logistic regression analysis of the variables associated with fractures, low BMD, and reduced TBS was conducted using stepwise backward adjusted models. The Wald test was conducted to estimate the significance of each variable in the model. The odds ratio (OR) and the 95% confidence interval (CI) are presented, and *P* < 0.05 indicated statistical significance.

## Results

### Baseline characteristics

Between March 2019 and March 2021, we retrospectively reanalyzed data from 85 patients with HFrEF and 142 controls who had previously had DXA scans.

The mean age of the HFrEFG was 65.1 ± 13.1 years (43.5% women). The HFrEFG had higher CCI than the CG, almost 80% had hypertension and dyslipidemia, one-third had diabetes mellitus, 11.8% were current smokers, two-thirds had a history of falls, and their mean BMI was 27.2 ± 4.6 kg/m^2^. The majority were physically active (85.9%), 62.4% NYHA II, without important functional limitation. Patients were under optimum evidence-based guideline treatment with 98% using beta blockers, 91.1% angiotensin-converting enzyme inhibitors or angiotensin II receptor blockers, 71% statins, 59% spironolactone, 53% aspirin or loop diuretics, 31% coumarins, 14% thiazides, 19% nitrates, 18% digoxin, and 17% hydralazine. The HFrEFG had fewer current smokers and White people than the controls, and they were more active (*P* < 0.001). Table 1 shows the patients´ and controls´ baseline characteristics.

**Table 1:** Baseline characteristics of the patients and controls.

Characteristics	HFrEFG	CG	*P* value
	*n* = 85	*n* = 142	
**Age (yr)**	65.1 ± 13.1	64.9 ± 11.3	0.65
**Gender**			0.9
Men–no. (%)	48 (56.5	78 (54.9	
Women–no. (%)	37 (43.5	64 (45.1	
**Ethnicity**			
White–no. (%)	45 (52.9)	139 (97.8)	< 0.001
**Weight (kg)**	71.4 ± 14.9	73.3 ± 11.8	0.15
**Height (m)**	1.62 ± 0.09	1.63 ± 0.1	0.33
**BMI (kg/m** ^**2**^ **)**	27.19 ± 4.62	27.4 ± 3.14	0.53
**Smoking–no. (%)**			
Current	10 (11.8)	65 (45.8)	< 0.001
Previous smoking	37 (49.3)	NA	
**Previous alcohol intake–no. (%)**	5 (5.9)	NA	
**Comorbidities–no. (%)**			
Arterial hypertension	67 (78.8)	33 (23.2)	< 0.001
Dyslipidemia	69 (81.2)	26 (18.3)	< 0.001
Diabetes mellitus	25 (29.4)	9 (6.3)	< 0.001
Cerebrovascular disease	25 (29.4)	0	< 0.001
Atrial fibrillation	19 (22.4)	NA	
COPD / Pneumopathies	12 (14.1)	0	< 0.001
**CCI (min.-max.)**	4.3 ± 1.55 (1–8)	2.1 ± 1.1 (0–4)	< 0.001
**Ground-level fall–no. (%)**	54 (63.5%)	NA	
**NYHA–no. (%)**			
I	9 (10.6)	NA	
II	53 (62.4)	NA	
II	23 (27)	NA	
**Physical activity level–no. (%)**			< 0.001
Sedentary	1 (1.2)	25 (17.6)	
Insufficiently active	11 (12.9)	24 (16.9)	
Active	73 (85.9)	28 (19.7)	
**LV diastolic dimension (mm)**			
Men (2-SD range: 42–58.4 mm)	61.5 ± 8.3	NA	
Women (2-SD range: 37.8–52.2 mm)	60.3 ± 8.9	NA	
**LV mass index (g/m** ^**2**^ **)**			
Men (NR: 50–102 g/m^2^)	146.4 ± 38.6	NA	
Women (NR: 40–88 g/m^2^)	156.1 ± 37	NA	
**LVEF (%)**	32.98 ± 6.33	NA	
**Right-ventricle enlargement–no. (%)**	32 (37.6)	NA	
**Right-ventricle systolic disfunction–no. (%)**	14 (16.47)	NA	
**PASP (mmHg)–RV: < 35 mmHg**	43.4 ± 11.1	NA	

HFrEFG = heart failure with reduced left ventricle ejection fraction group; CG = control group; Yr = years old; COPD = chronic obstructive pulmonary disease; CCI = Charlson Comorbidity Index; no. = number; (%) = percentage; NYHA = New York Heart Association functional capacity classification; BMI = body mass index; kg = kilogram; m = meters; LV = left ventricle; LVEF = left ventricle ejection fraction; PSAP = echocardiography estimated pulmonary artery systolic pressure; mmHg = millimeters of mercury; NA = not available; SD = standard deviation; NR = normal range of left ventricle mass index; RV: reference value.

BMI, weight, height, and biochemistry exams were similar between the 2 groups, except for creatinine, which was higher in the HFrEFG (1.13 ± 0.34 vs 0.99 ± 0.32, *P* < 0.001). The minimum and maximum value of N-terminal pro B-type natriuretic peptide (NT-proBNP) of the HFrEFG were 181 and 9.901 pg/mL (Tables 1 and 2).

**Table 2:** Laboratory exams presented by patients and controls.

Laboratory exams	HFrEFG	CG	*P* value
	*n* = 85	*n* = 142	
**Sodium (mEq/L)**	138 ± 3.46	NA	
**Potassium (mEq/L)**	4.48 ± 0.72	NA	
**Urea (mg/dL)**	45.3 ± 20	NA	
**Creatinine (mg/dL)**	1.13 ± 0.34	0.99 ± 0.32	< 0.001
**TSH (μUI/mL)**	2.58 ± 3.41	2.23 ± 1.06	0.23
**Free T4 (pg/dL)**	1.07 ± 0.31	NA	
**Albumin (g/dL)**	4 ± 0.33	4.09 ± 0.55	0.23
**25 OH vitamin D (ng/mL)**	27.1 ± 15.2	27.6 ± 14.04	0.89
**Total calcium (mg/dL)**	8.67 ± 2.4	9.43 ± 1.24	0.14
**Inorganic phosphate (mg/dL)**	3.34 ± 1.1	NA	
**C-reactive protein (mg/L)**	0.51 ± 0.94		
**Hemoglobin (g/dL)**	13.81 ± 1.72	14.09 ± 2.72	0.24
**Leukocytes × 10** ^**9**^ **/L**	7.541 ± 2.473	NA	
**HbA1c (%)**	5.25 ± 1.91	NA	
**INR**	0.89 ± 1.3	NA	
**HDL (mg/dL)**	38 ± 16.2	NA	
**c-LDL (mg/dL)**	92.5 ± 44.1	NA	
**Triglycerides (mg/dL)**	108 ± 61.5	NA	
**Total Cholesterol (mg/dL)**	151.7 ± 61.1	NA	
**NT-proBNP (pg/mL)**	821 ± 1.724	NA	

TSH = thyroid stimulant hormone; Free T4 = free form of thyroxine; 25 OH vitamin D = 25 hydroxyvitamin D; HbA1c% = hemoglobin glycosylated; INR = international normalized ratio; HDL = high-density lipoprotein density; c-LDL = low density lipoprotein; NT-proBNP = N-terminal pro B-type natriuretic peptide; NA = not available.

In the HFrEFG, the mean daily intake of calories, calcium, and vitamin D were 1472.8 ± 537.4 Kcal, 469.9 ± 261.65 mg, and 2.68 ± 2.1 μg, respectively, with most members having insufficient intake of calories (65.8%), calcium (95.3%), and vitamin D (100%).

### BMD analysis

BMD was similar in the two groups in all sites, with 35% of the HFrEFG having OP and 22% osteopenia with no difference compared to the CG (Table 3).

**Table 3:** Bone mineral density (BMD), trabecular bone score (TBS), body composition, strength, and performance in patients and controls.

Variables	HFrEFG	CG	*P* value
	*n* = 85	*n* = 142	
**Lumbar spine BMD (g/m** ^**2**^ **)**			
Men	1.024 ± 0.2	1.26 ± 0.23	0.21
Women	1.08 ± 0.23	1.04 ± 0.21	0.28
**Femoral neck BMD (g/m** ^**2**^ **)**			
Men	0.99 ± 0.19	0.94 ± 0.13	0.92
Women	0.92 ± 0.21	0.84 ± 0.24	0.38
**Total hip BMD (g/m** ^**2**^ **)**			
Men	1.08 ± 0.18	0.03 ± 0.14	0.75
Women	0.98 ± 0.21	0.9 ± 0.14	0.71
**Low BMD–no. (%)**	49 (57.6)	86 (60.6)	0.91
**TBS**	1.296 ± 0.14	1.320 ± 0.11	0.07
Reduced TBS. (%)	44 (51.8)	65 (46.1)	0.12
Partially degraded TBS–no. (%)	20 (23.5)	38 (27)	0.6
Degraded TBS–no. (%)	24 (28.2)	27(19)	0.14
**TBS (BMI 15–37 kg/m**^**2**^**)** ^**a**^	1.293 ± 0.14	1.320 ± 0.11	0.03
**TBS (w/o diabetes)** ^**b**^	1.286 ± 0.15	1.330 ± 0.11	0.14
**TBS (with normal BMD)** ^**c**^	1.342 ± 0,14	1.396 ± 0.67	0.04
**Reduced TBS with normal BMD–no. (%)** ^**d**^	11 (30.5)	9 (16)	0.87
**ALM (kg)**			
Men	22.8 ± 3.82	22.38 ± 3.19	0.55
Women	15.6 ± 2.3	15.35 ± 2.3	0.62
**ALM/h** ^**2**^			
Men	8.14 ± 0.94	7.82 ± 0.81	0.06
Women	6.4 ± 0.88	6.22 ± 0.69	0.29
**TLM (kg)**			
Men	51.78 ± 7.82	50.9 ± 5.6	0.94
Women	37.06 ± 4.74	36.9 ± 4.6	0.89
**TFM (kg)**			
Men	21.96 ± 8.21	23.7 ± 6.7	0.06
Women	25.1 ± 9.75	27.6 ± 6.4	0.16
**Trunk Fat mass (kg)**			
Men	13.46 ±0.76	NA	
Women	12. 69 ± 0.93	NA	
**Handgrip strength (kg)**			
Men	33.5 ± 8.34	35.31 ± 3	0.17
Women	20.04 ± 5.54	31.08 ± 3.1	< 0.001
**Gait speed (m/s)**	0.98 ± 0.27	NA	

HFrEFG = heart failure with reduced left ventricle ejection fraction group; CG = control group; BMD = bone mineral density; w/o = without; TBS = trabecular bone score; Low TBS = degraded or partially degraded; ALM = appendicular lean mass; ALM/h^2^ = appendicular lean mass adjusted to the square of the height; TFM = total fat mass; NA = not available.

a: 03 patients were excluded from HFrEFG and none from CG.

b: 25 patients with T2DM were excluded from HFrEFG and 09 from CG.

c: 49 patients were excluded from HFrEFG and 86 from CG.

d: absolute number and its percentage only in those who had normal BMD.

Compared to those with normal BMD, HFrEFG patients with low BMD were older (*P* = 0.005) and had higher CCI (*P* = 0.03) and urea (*P* = 0.04); worse functional capacity (*P* = 0.008); and lower weekly physical activity (*P* = 0.02), weight (*P* < 0.001), height (*P* = 0.02), BMI (*P* = 0.001), handgrip strength in men (*P* = 0.04), gait speed (*P* = 0.02), hemoglobin (*P* = 0.003), globular volume (*P* = 0.002), lean mass (*P* < 0.005), trunk fat mass in women (*P* = 0.03), and TBS (*P* = 0.008) (Table 4).

**Table 4:** Comparative analysis of the variables associated with low bone mineral density (BMD) in patients with HFrEF.

Variables	HFrEFG with low BMD	HFrEFG with normal BMD	*P* value
	*n* = 49	*n* = 36	
**Age (yr)**	68.6 ± 11.2	60.3 ± 14.1	0.005
**CCI**	4.61 ± 1,48	3.81 ± 1.55	0.03
**Functional capacity**			0.008
NYHA I–no. (%)	1 (2.04)	8 (22.2)	
NYHA II–no. (%)	32 (65.3)	21 (58.3)	
NYHA III–no. (%)	16 (32.65)	7 (19.4)	
**Physical activity**			
IPAQ–Mets.min/week	2,014.1 ± 1,814.1	3,536.2 ± 3,688.2	0.02
**Weight (kg)**	65.7 ± 11.9	79.07 ± 15.1	< 0.001
**Height (m)**	1.6 ± 0.08	1.65 ± 0.09	0.02
**BMI (kg/m** ^**2**^ **)**	25.76 ± 3.9	29.1 ± 4.9	0.001
**Handgrip strength (kg)**			
Men	30.99 ± 6.34	35.90 ± 9.4	0.04
Women	19.12 ± 5.5	22.20 ± 5.2	0.12
**Gait speed (m/s)**	0.95 ± 0.3	1.04 ± 0.25	0.02
**Hemoglobin (g/dL)**	13.35 ± 1.7	14.45 ± 1.6	0.003
**Globular volume (%)**	40.60 ± 4.5	43.80 ± 4.3	0.002
**Urea (mg/dL)**	49.08 ± 22.3	40.30 ± 16.97	0.04
**ALM (kg**			
Men	21.04 ± 2.7	24.38 ± 4.1	0.005
Women	14.80 ± 1.9	17.50 ± 2.1	0.002
**ALM/h**^**2**^ **(kg/m**^**2**^**)**			
Men	7.65 ± 0.6	8.59 ± 0.98	< 0.001
Women	6.11 ± 0.7	7.10 ± 0.89	0.005
**TLM (kg)**			
Men	48.50 ± 5.4	54.80 ± 8.6	0.01
Women	35.40 ± 3.9	40.90 ± 4.4	0.002
**Trunk fat mass (kg)**			
Men	12.28 ± 3.9	14.53 ± 6.3	0.14
Women	11.29 ± 5.2	16.00 ± 5.5	0.03
**Total mass (kg)**			
Men	72.60 ± 37.	81.50 ± 41.4	0.01
Women	62.40 ± 32.5	86.20 ± 40.3	0.004
**TBS**	1.262 ± 0.13	1.342 ± 0.14	0.008
**TBS (BMI 15–37 kg/m**^**2**^**)** ^**a**^	1.265 ± 0.12	1.333 ± 0.13	0.025
**TBS (no DM2)** ^**b**^	1.242 ± 0.14	1.352 ± 0.14	0.005

HFrEFG = heart failure with reduced left ventricle ejection fraction group; CG = control group; BMD = bone mineral density; TBS = trabecular bone score; Yr = years old; CCI = Charlson Comorbidity Index; IPAQ: International Physical Activity Questionnaire; Mets = metabolic equivalents; NYHA = New York Heart Association functional capacity classification; kg = kilogram; m = meters; BMI = body mass index; ALM = appendicular lean mass; ALM/h^2^ = appendicular lean mass adjusted to the square of the height; TLM = total lean mass; TBS = trabecular bone score. DM2 = type 2 diabetes

a: 03 patients of the entire HFrEF group were excluded.

b: 25 patients of the entire HFrEF group were excluded.

After multivariate logistic regression analysis, only BMI (OR 0.81; 95% CI 0.7–0.94; *P* = 0.007) and hemoglobin (OR 0.7; 95% CI 0.5–0.98; *P* = 0.04) were associated with low BMD.

### TBS analysis

**Fig 1:**
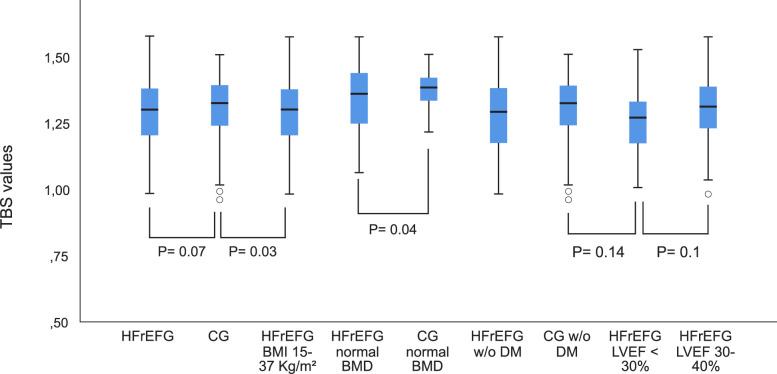
Comparison of trabecular bone score (TBS) values between patient and controls and in subgroups according to body mass index (BMI), normal bone mineral density (BMD), diabetes mellitus (DM) and ventricular ejection fraction (LVEF). Comparisons of TBS values between groups and in subgroups are shown and delimited by square brackets below the corresponding box plot. TBS = trabecular bone score; HFrEFG = heart failure with reduced left ventricle ejection fraction group; CG = control group; HFrEFG BMI 15–37 Kg/m^2^ = HFrEFG patients who had a body mass index between 15–37 Kg/m^2^; HFrEFG normal BMD = HFrEFG patients who had normal bone mineral density; CG normal BMD = controls who had normal bone mineral density; HFrEFG w/o DM = HFrEFG patients who did not have type 2 diabetes mellitus; CG w/o DM = controls who did not have type 2 diabetes mellitus; HFrEFG LVEF < 30% = HFrEFG patients who had left ventricular ejection fraction < 30%; HFrEFG LVEF 30–40% = HFrEFG patients who had left ventricular ejection fraction between 30%-40%. Notes: 1) Three patients were excluded of the HFrEFG to compose the HFrEF BMI 15–37 kg/m^2^ subgroup and none of the controls were in this range; 2) 49 patients with low bone mass, osteoporosis or osteopenia were excluded from HFrEFG and 86 from CG; 3) 25 patients with type 2 diabetes mellitus were excluded from HFrEFG and 09 from CG.

Fifty-two percent of the HFrEFG had reduced TBS, which was not significantly different from the 46% seen in the controls (*P* = 0.69), even when we excluded patients with DM2. However, when the BMI was between 15 and 37 kg/m^2^, the HFrEFG had lower TBS than the CG (*P* = 0.03) at similar BMI values (27.02 ± 3.97 kg/m^2^ in the HFrEFG vs 27.4 ± 3.14 kg/m^2^ in the CG, *P* = 0.25). In addition, patients with normal BMD had lower TBS values (1.342 ± 0.14) than the controls (1.396 ± 0.67, *P* = 0.04). The mean TBS did not differ accordingly with the ejection fraction (1.256 ± 0.13 in LVEF < 30% vs 1.310 ± 0.14 in LVEF 30–40%; *P* = 0.1), but there was a larger proportion rate of reduced TBS in patients with LVEF < 30% (70% vs 43%; *P* = 0.02). Fig 1 shows the TBS values in the study´s whole groups and subgroups.

Compared to those with normal TBS, patients who had reduced TBS (1.192 ± 0.098) in the HFrEFG were older (*P* = 0.002), and had higher CCI (*P* = 0.014), left ventricle mass index (*P* = 0.009), and estimated pulmonary artery systolic pressure (*P* = 0.05). Conversely, they had lower albumin (*P* = 0.01), hemoglobin (*P* = 0.009), globular volume (*P* = 0.024), use of nitrates (*P* = 0.05), statins (*P* = 0.011), and vitamin D intake (*P* = 0.023). Also, the men had lower handgrip strength (*P* = 0.013) and the women had a lower percentage of trunk fat mass (*P* = 0.047) (Table 5).

**Table 5:** Comparative analysis of the variables associated with reduced TBS in patients with HFrEF.

Variables	HFrEFG with reduced TBS	HFrEFG with normal TBS	*P* value
	*n* = 44	*n* = 41	
**Age (yr)**	69.5 ± 9.9	60.4 ± 14.5	0.002
**CCI**	4.68 ± 1.33	3.83 ± 1.67	0.01
**HGS (kg)**			
Men	29.87 ± 10.2	35.96 ± 7.79	0.01
Women	19.72 ± 5.98	20.64 ± 4.78	0.61
**Albumin (g/dL)**	3.9 ± 0.38	4.1 ± 0.23	0.01
**Hemoglobin (g/dL)**	13.3 ± 1.6	14.3 ± 1.7	0.009
**Globular volume (%)**	40.8 ± 4.6	43.2 ± 4.5	0.02
**Low ALM–no. (%)** ^**a**^	20 (45.5)	9 (22)	0.025
**Trunk fat mass (%)**			
Men	29.65 ± 15.6	30.5± 16.2	0.12
Women	40.9 ± 21.6	48.4 ± 18.2	0.04
**Left ventricular mass index (g/m** ^**2**^ **)**	159.17 ± 37.37	141.39 ± 36.95	0.009
**Statin use–no. (%)**	26 (59.1)	34 (82.9)	0.01
**PASP (mmHg)**	41 ±11.7	45.4 ± 10.4	0.05
**Nitrates use–no. (%)**	12 (27.3)	4 (9.8)	0.05
**Consumed energy intake (%)**	80.66 ± 26.2	68.7 ± 29.1	0.02
**Vitamin D intake (μg)**	3.2 ± 2.4	2.1 ± 1.5	0.02

HFrEFG = heart failure with reduced left ventricle ejection fraction group; CG = control group; TBS = trabecular bone score; Yr = years-old; CCI = Charlson Comorbidity Index; HGS = handgrip strength; ALM = appendicular lean mass; PSAP = echocardiography estimated pulmonary artery systolic pressure; mmHg = millimeters of mercury.

a: low ALM = < 19.75 kg in men and < 15.02 in women.

After multivariate logistic regression analysis, age (OR 1.05; 95% CI 1.01–1.10; *P* = 0.026), albumin (OR 0.12; 95% CI 0.01–0.96; *P* = 0.046), statin use (OR 0.27; 95% CI 0.08–0.84; *P* = 0.03), and percentage of consumed energy intake (OR 1.03; 95% CI 1.01–1.05; *P* = 0.014) were associated with reduced TBS in the HFrEFG.

The correlations between TBS and BMD of the spine, femoral neck, and total hip were moderate (*r* = 0.56, 0.51, and 0.56, respectively; *P* < 0.001), and TBS and BMI were weakly correlated (*r* = 0.28; *P* = 0.01).

### Fractures

In the HFrEFG, 14 patients had a history of low-impact fractures (16.5%), and VFs were seen in 36 (42.4%) on VFA (kappa = 0.11, *P* = 0.2), with thoracic vertebra T8 being more frequently fractured on VFA (25% of all fractures). Fractures were not associated with past falls (48.1% in fallers vs 32.3% in non-fallers, *P* = 0.2) or BMD classification (41.7% of fractures on VFA occurred in normal BMD, 36.8% in osteopenia, and 46.7% in OP), and TBS did not affect the presence of fractures (*P* = 0.7). After multivariate logistic regression analysis, fractures on VFA were associated with older age (68.3 ± 12.9 vs 62.8 ± 12.8, OR 1.05, 95% CI 1.001–1.11, *P* = 0.04), higher LVEF (33.5 ± 6.1% vs 32.35 ± 6.2%, OR 1.14, 95% CI 1.02–1.26, *P* = 0.02), and use of hydralazine (27.8% vs 8%, OR 8, 95% CI 1.64–39.2, *P* = 0.01).

### FRAX evaluation and fracture risk

**Fig 2:**
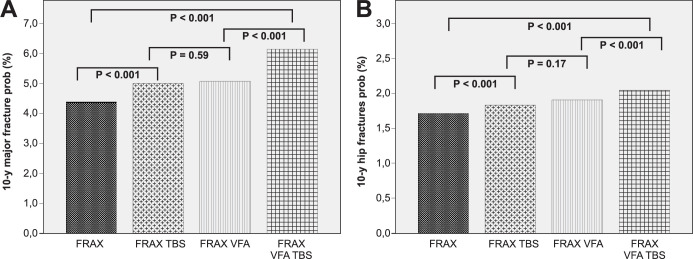
Comparison of Fracture Risk Assessment Tool (FRAX) results before and after inclusion of fractures on vertebral fracture assessment (VFA), trabecular bone score (TBS), and both parameters for major and hip fracture risk in patients with HFrEF. Comparisons between the 10-year major fracture risk (A) and hip fracture risk (B) are shown in square brackets. FRAX: probability of fractures estimated by the Fracture Risk Assessment Tool considering clinical data and bone mineral density; FRAX VFA: Probability of 10-year fractures estimated by the FRAX tool plus the accounting of subclinical fractures diagnosed by the Vertebral Fracture Assessment (VFA); FRAX TBS: Probability of 10-year fractures estimated by the FRAX tool adjusted by TBS; FRAX VFA TBS: Probability of 10-year fractures estimated by the FRAX tool considering the concomitant adjustment by TBS and the accounting of subclinical fractures by VFA.

The FRAX showed a 10-year risk of 4.38 ± 4.04 for major osteoporotic fractures and 1.71 ± 2.9 for hip fractures in the HFrEFG. The adjustment by TBS and the inclusion of subclinical fractures diagnosed on VFA increased the 10-year risk of fractures to 4.99 ± 4.5 and 5.09 ± 4.5 for major fractures, and to 1.77 ± 3 and 1.91 ± 3.15 for hip fractures. When both were considered concurrently, they increased the 10-year risk of major and hip fractures to 5.7 ± 4.9 and 2.03 ± 3.26, respectively. Fig 2 shows the comparison of major (A), and hip (B) fracture probabilities assessed using the FRAX with adjustments for TBS, inclusion of fractures on VFA, and both.

## Discussion

This study showed that patients with HFrEF had poor bone quality, with low baseline TBS values that were lower than those of the controls in the presence of normal BMD and when BMI was adequate for densitometry analysis. Also, we showed that some variables related to prognosis, severity, and treatment of HF were associated with reduced TBS, fractures, and low BMD and that the TBS adjustment as well as the active search for subclinical fractures by the VFA increased the prediction of the risk of subsequent fractures by approximately one-fifth, with a cumulative effect of both, emphasizing TBS as an additive contributor for fracture risk evaluation.

The prevalence of OP was close to that Leslie et al. found in the general population of the Manitoba bone density program [27], although we found a higher prevalence of OP and a lower prevalence of osteopenia than those previously reported in HF [10, 11, 28–30]. In all likelihood, the difference arose due to our patient´s older age, long time of treatment, and follow-up as well as the number of comorbidities that is common in HF and possibly caused additional bone damage. Also, the proportion of men versus women in our sample could have influenced the OP prevalence because studies in HF have traditionally included a larger proportion of men [1, 2, 10–16, 19, 21, 29, 30]. The lack of difference in BMD between the patients and controls cannot be explained only by the relatively small sample size or the pairing of the HFrEFG and the controls. Our patients were more physically active, had similar lean mass, and had even proportionally greater lean mass than that of the controls. In addition, the controls were predominantly White, with potentially higher income and less mandatory daily physical activity than the patients. All of these aspects may have mitigated possible comparative between-group differences in the BMD analysis.

Our BMD findings agree as to which one has been classically described in the general population, with older age and lower BMI being risk factors for OP and fractures [1, 2, 29].Moreover, we found that other markers and risk factors for bone disease, such as comorbidities, lower amount of physical activity, worse physical performance, lower lean mass, handgrip strength (in men), and trunk fat mass (in women), were all also associated with low BMD, supporting the scarce studies that took these variables into account [1, 2, 12, 29, 30].

Patients with low BMD would also be expected to have low TBS values [27, 31]. However, the greatest TBS value and its expected contribution are related to the prediction of fractures when BMD is not yet in the osteoporotic range [32], especially considering that TBS is an independent predictor of fractures [18, 33]. Precisely for this reason, we believe that this study´s major contribution was the demonstration that patients with HFrEF and normal BMD had lower TBS than the controls, adding information in the evaluation of risk of fracture in this population. Another important observation was the exclusion of BMI extremes to avoid misinterpretation of TBS, knowing that adipose tissue is a confounding factor in the quantitative assessment of TBS [28, 34], and the weak correlation between TBS and BMI found in this study highlights this issue. In addition, FRAX results adjusted by TBS showed similar impact in the fracture risk estimation as the inclusion of fractures observed using VFA. Past fractures are known to be the greatest fracture risk predictor [17, 35], and the fracture prediction increased with the active searching of vertebral fractures (VFs) by VFA. Therefore, detailed investigation of subclinical fractures by VFA or even X-ray, in addition to the evaluation of bone quality, can make a difference in clinical practice. Importantly, we found a stronger correlation between TBS and BMD (0.51 to 0.56) than observed in the Manitoba Study (0.26 to 0.33) [33], which adds to the importance of TBS as an early discriminator for assessing fracture risk over time.

The HFrEFG had low baseline TBS values, and the pairing with controls may have also attenuated their between-group difference. Patients who had severely reduced LVEF (<30%) had even lower TBS, and there was a higher percentage of reduced TBS in the HFrEFG than in the CG. Epidemiological variables of HF, such as age, comorbidities, higher left ventricular mass, pulmonary artery systolic pressure, and the use of nitrate, which is commonly used in association with hydralazine in difficult-to-treat patients [36], were also associated with reduced TBS.

As interesting as the association of lower values of Hb/globular volume with low BMD was, there was also an association of lower values of albumin and Hb/globular volume with reduced TBS, the former having a prognostic role in the mortality of the elderly, in addition to the association with malnutrition, comorbidities, inflammatory cytokines, and sarcopenia [37]. The latter is important as a marker of chronic disease and as the prognostic and symptomatic variable of HF, being part of the PREDICT-HF model [38], as well as lower levels of albumin and higher urea levels, which were also associated with low BMD in this study. We also observed the association of reduced TBS with weakness in men, a mainstay pillar of sarcopenia diagnoses.

Lower rates of statin use were also associated with reduced TBS in the HFrEFG, a finding that had not been demonstrated in this population. Studies have associated the use of statins with higher BMD and with lower rates of OP [39] and VFs [17]. On the other hand, there is also evidence of a negative correlation of BMD with hypercholesterolemia, as well as positive correlations of BMD with metabolic syndrome and hypertriglyceridemia [40, 41], but none of these studies evaluated TBS. Based on the data of the statin–BMD associations, our findings regarding statin use and TBS agree with a possible statins bone protective effect.

Almost a third of the HFrEF patients in this study had diabetes, and because diabetic patients are at increased risk of fractures even with normal or even increased BMD, assessment of bone quality is very important. Bone damage related to diabetes can be explained in a multifactorial way and can occur through neuroendoinflammatory mechanisms, the production of hydrogen sulfide (H2S), hypoglycemia, deficient muscle activation, falls, or even the action of antidiabetic medications [42]. To reduce diabetes-related bias and increase our findings´ specificity, we excluded all diabetics from a statistical subanalysis of TBS, which demonstrated no significant differences. Our exclusion criteria eliminated patients with severe complications who were decompensated or difficult to treat and control, including diabetics who used insulin, avoiding important biases. Therefore, we understand that diabetes was probably not a confounding factor in the interpretation of our findings.

Our prevalence of clinical VFs in HFrEF was close to that Lyons et al. found in HF in Canada [17], with most of the VFs happening in osteoporotic patients although Rakusa and coworkers observed it in patients with osteopenia with impaired TBS [19]. However, neither of these studies associated TBS and BMD with fractures, maybe because of the lack of sample power or because the baseline TBS values were already low. Because fractures occur in patients with OP and osteopenia, BMD is not the only factor to be considered in the risk of fractures [43, 44]. Indeed, previous VF is an important risk factor to be considered for subsequent OP fractures [17], as our study shows, in which 40% of the HFrEFG patients who had normal BMD also presented with VFs.

Patients with HF, especially those who are more symptomatic and with worse LV systolic dysfunction, have lower non-vertebral BMD [2, 12, 14] and more fractures [3], but the unequivocal association between the severity of the reduction in LVEF and the presence of osteoporotic VFs has not been demonstrated. We considered that the negative association between LVEF with VFs possibly arose due to chance, especially considering the similarity of the LV systolic dysfunction between patients who had and those who did not have any VF. On the other hand, the use of hydralazine, which is a known marker of HF severity and more symptomatic disease [36, 45], was also associated with VF in this work and indirectly reflects the severity of the HFrEF.

Qualitative and quantitative aspects of diet affect bone health and quality [46, 47], and the participants in our study sample had a poor diet quality. We found that a percentage increase in the daily ingested calories was associated with a reduction in TBS in HFrEFG, independent of BMI. Interestingly and inversely to the TBS results, lower calorie intake was associated with VF, which could potentially be explained by the scenarios associated with bone involvement. Patients who already had fractures are expected to have more advanced bone and heart disease, which could be associated or not with diet, whereas in the subgroup of patients with HFrEF and reduced TBS, the association of TBS values with the percentage of energy intake could also be related to the quality of the diet and/or to an early onset of bone changes.

This study´s limitations were the unicentric site; the cross-sectional design; the difficulties related to the heart disease severity, which limited the highly judicious selection of the patients; the relatively small; and the stratification of subgroups analysis, which further reduced the number of statistical comparisons. Potential memory bias of past fractures may have also occurred.

This study´s advantages were that we evaluated the bone involvement in very well-treated chronic heart disease, for which there is still a lack of data in the literature, mainly related to TBS. In addition, patients were stable for a long time and were highly physically active, and no major confounding factors related to the heart transplant setting, such as corticosteroid and calcineurin–calmodulin inhibitor use, were present, which increased our findings´ specificity, although there were many differences related to many comorbidities in HFrEFG, which is inherent to the profile of patients with HF. Further, to maintain data specificity, we chose not to compare the BMD data with the population reference values (Z and T scores). Also, the HFrEFG had no significant presence of edema, reinforcing the densitometric analysis and reducing possible interferences of its accurate measurement.

In conclusion, our results showed that patients with HFrEF had poor bone quality, with a better discriminating impact of the TBS assessment when BMD was normal and BMI was suitable for densitometric analysis. Some variables related to energy intake and to the prognosis, severity, and treatment of HFrEF were associated with reduced TBS, fractures, and low BMD, and the TBS adjustment as well as the active search for subclinical fractures by the VFA increased the prediction of the risk of fractures by approximately one-fifth, with a cumulative effect of both.
