# Correction: Bone quality, mineral density, and fractures in heart failure

**DOI:** 10.1371/journal.pone.0353220

**Published:** 2026-07-06

**Authors:** Andre Luiz Canteri, Luana Bassan Gusmon, Cesar Luiz Boguszewski, Victoria Zeghbi Cochenski Borba

In Fig 1, the comparison among the subgroups of the fourth bracket is incorrect. The fourth bracket should pertain to the comparison between the TBS value (y-axis) of the subgroups HFrEFG w/o DM and CG w/o DM. Please see the correct Fig 1 here.

**Fig 1:**
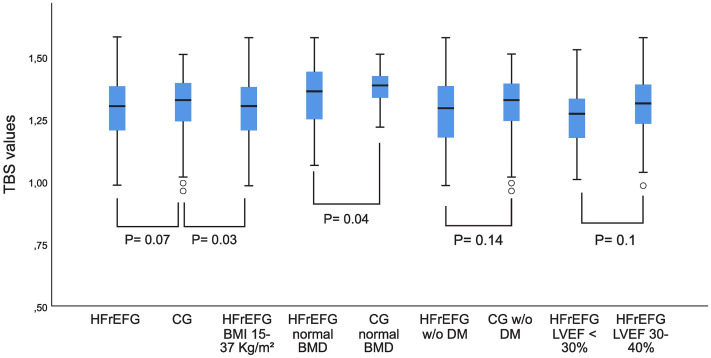
Comparison of trabecular bone score (TBS) values between patient and controls and in subgroups according to body mass index (BMI), normal bone mineral density (BMD), diabetes mellitus (DM) and ventricular ejection fraction (LVEF). Comparisons of TBS values between groups and in subgroups are shown and delimited by square brackets below the corresponding box plot. TBS = trabecular bone score; HFrEFG = heart failure with reduced left ventricle ejection fraction group; CG = control group; HFrEFG BMI 15–37 Kg/m2 = HFrEFG patients who had a body mass index between 15–37 Kg/m2; HFrEFG normal BMD = HFrEFG patients who had normal bone mineral density; CG normal BMD = controls who had normal bone mineral density; HFrEFG w/o DM = HFrEFG patients who did not have type 2 diabetes mellitus; CG w/o DM = controls who did not have type 2 diabetes mellitus; HFrEFG LVEF < 30% = HFrEFG patients who had left ventricular ejection fraction < 30%; HFrEFG LVEF 30–40% = HFrEFG patients who had left ventricular ejection fraction between 30%-−40%. Notes: 1) Three patients were excluded of the HFrEFG to compose the HFrEF BMI 15–37 kg/m2 subgroup and none of the controls were in this range; 2) 49 patients with low bone mass, osteoporosis or osteopenia were excluded from HFrEFG and 86 from CG; 3) 25 patients with type 2 diabetes mellitus were excluded from HFrEFG and 09 from CG.
